# Diversity of the lysozyme fold: structure of the catalytic domain from an unusual endolysin encoded by phage Enc34

**DOI:** 10.1038/s41598-022-08765-1

**Published:** 2022-03-23

**Authors:** Elina Cernooka, Janis Rumnieks, Nikita Zrelovs, Kaspars Tars, Andris Kazaks

**Affiliations:** 1 Latvian Biomedical Research and Study Centre, Ratsupites 1 k-1, Riga, 1067 Latvia; 2 Faculty of Biology, University of Latvia, Jelgavas 1, Riga, 1004 Latvia

**Keywords:** Molecular biology, Structural biology

## Abstract

Endolysins are bacteriophage-encoded peptidoglycan-degrading enzymes with potential applications for treatment of multidrug-resistant bacterial infections. *Hafnia* phage Enc34 encodes an unusual endolysin with an N-terminal enzymatically active domain and a C-terminal transmembrane domain. The catalytic domain of the endolysin belongs to the conserved protein family PHA02564 which has no recognizable sequence similarity to other known endolysin types. Turbidity reduction assays indicate that the Enc34 enzyme is active against peptidoglycan from a variety of Gram-negative bacteria including the opportunistic pathogen *Pseudomonas aeruginosa* PAO1. The crystal structure of the catalytic domain of the Enc34 endolysin shows a distinctive all-helical architecture that distantly resembles the α-lobe of the lysozyme fold. Conserved catalytically important residues suggest a shared evolutionary history between the Enc34 endolysin and GH73 and GH23 family glycoside hydrolases and propose a molecular signature for substrate cleavage for a large group of peptidoglycan-degrading enzymes.

## Introduction

The increasing prevalence of multidrug-resistant bacteria is being recognized as a major threat to global public health^1^. While antibiotic resistance has existed for at least several hundred million years as a defense measure in an ongoing ‘chemical warfare’ in the microbial world^2^ and a diverse set of antimicrobial resistance mechanisms have been present in nature for a very long time^3^, the current practice of excessive and often poorly substantiated medical and agricultural antibiotic use has greatly accelerated the evolutionary drive for selecting antibiotic-resistant bacteria^4^. With the number of newly discovered antibiotics dwindling, development of potential alternative treatments such as natural and synthetic antimicrobial peptides^5^, antibacterial monoclonal antibodies^6^, and bacteria-killing viruses (bacteriophages or phages)^7^ is of high priority to avoid a global health crisis caused by life-threatening and untreatable bacterial infectious diseases.

Phage therapy—the potential use of bacteriophages for controlling bacterial infections—was recognized already in the early twentieth century but has not seen significant developments due to unclear regulatory frameworks^8^, challenges in large-scale phage propagation, issues such as development of phage-resistant bacteria and anti-phage immune response, and even risks of horizontal gene transfer^9^. The antibacterial properties of phages, however, can be to a large extent reduced to the action of their endolysins, viral peptidoglycan-degrading enzymes which at the end of the replication cycle digest the bacterial cell wall, leading to osmotic rupture of the cell and release of viral progeny in the environment^10^. Purified endolysin preparations have demonstrated notable antimicrobial activity^11^ and, in contrast to conventional antibiotics, endolysins are extremely slow to induce resistance^12^. These enzymes can be further modified to alter their host range and enzymatic properties^13^, and they can be generally easily produced in industrial-scale quantities.

Despite achieving the same function of peptidoglycan cleavage, bacteriophage endolysins have markedly different structure and underlying mechanisms of action^14^. Endolysins from bacteriophages targeting Gram-positive bacteria usually have a modular structure of at least one enzymatically active domain (EAD) and one or more cell wall-binding domains (CBDs) while those from phages with Gram-negative hosts generally consist of only a single catalytic domain^15^ although some have additional N-terminal transmembrane domains^16^ or C-terminal amphipathic helices^17^. The EADs are remarkably diverse and belong to several functional classes that can target either glycosidic bonds (*N*-acetylglucosaminidases, *N*-acetylmuramidases and transglycosylases), the sugar-peptide linkage (*N*-acetylmuramoyl-l-alanine amidases), or the interpeptide bridge (various endopeptidases) and EADs of the different types can in turn adopt vastly different folds (reviewed by Broendum et al.^14^).

Phages are the most abundant and genetically diverse biological entities on Earth, and their gene pool appears to be a nearly limitless resource for a large variety of different proteins, including endolysins^18^. Therefore, despite the already documented vast variety of these enzymes, the overall structural and functional landscape of bacteriophage endolysins still remains incompletely explored and likely holds many more novel and useful enzymes for future clinical, industry and biotechnology applications. Here we report functional and structural characterization of such a new type of endolysin isolated from bacteriophage Enc34.

## Results

### Endolysin identification

Bacteriophage Enc34 is a siphovirus previously isolated and sequenced in our laboratory^19^. Enc34 is related to bacteriophages of the *Chivirus* genus that comprises mostly *Salmonella* phages of similar genome size and organization. Based on biochemical tests, the host bacterium of the Enc34 phage was initially identified as *Enterobacter cancerogenus*, but more recent molecular analysis has suggested that the host most likely is an unusual Bgl+ Pro− strain of *Hafnia alvei*. The genome of the Enc34 phage is approximately 60 kb long and contains 80 predicted open reading frames (ORFs), more than a half of which do not have an identifiable function.

**Figure 1:**
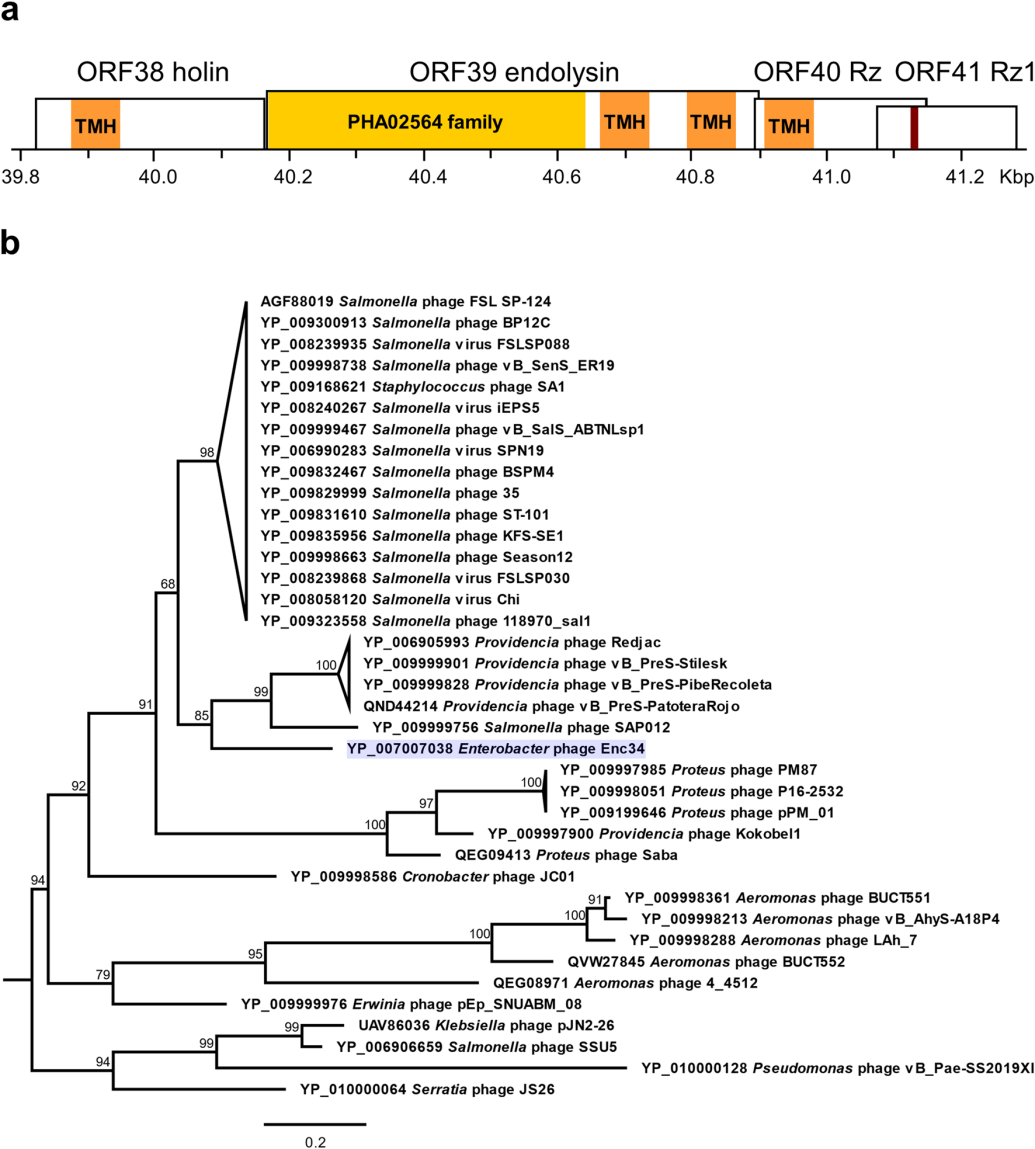
Endolysin proteins in Enc34 and related bacteriophages. (**a**) Organization of the lysis module of the Enc34 bacteriophage. Locations of the catalytic domain of the endolysin (PHA02564, yellow), transmembrane helices (TMH, orange) and a lipoprotein signal cleavage site (dark red) within the lysis proteins are indicated. (**b**) Phylogeny of Enc34-type endolysins. The tree is drawn to scale with branch lengths corresponding to the number of amino acid substitutions per site. Tip labels include NCBI accession numbers and corresponding phage names for the respective endolysin proteins. Ultrafast bootstrap support percentages are indicated adjacent to the nodes. For clarity, branches of some of the most populated and well-supported clades containing very similar sequences have been collapsed and are represented as triangles.

The virion morphogenesis module in the Enc34 genome is followed by a small cassette of four ORFs (Fig. 1a) which show no notable homology to any studied gene products, but which were predicted to comprise the lysis module of the phage by similarity with phage λ^20^. The first ORF within the module, ORF38, encodes a small ~ 12 kDa protein with a double-start motif and a predicted N-terminal transmembrane helix that resembles the λ holin. The last two gene products ORF40 and ORF41 are predicted to contain a transmembrane helix and a lipoprotein signal peptide, respectively, resembling the inner- and outer-membrane components Rz and Rz1 of the spanin complex of phage λ. However, contrary to λ, Enc34 ORF40 is not completely embedded in ORF41 but instead their sequences overlap as in phage P2^21^.

The remaining ORF in the cassette, ORF39, encodes a 26 kDa protein with an N-terminal domain of the PHA02564 family and a predicted C-terminal two-helix transmembrane domain. Based on its location in-between the other lysis genes, and in absence of any other candidates in the genome, the ORF39 was assumed to encode an endolysin of an apparently novel class of these enzymes. Proteins of similar sequence and predicted architecture are found also in genomes of other enterobacterial phages (Fig. 1b).

### Enzymatic activity

To enable functional and structural studies of the putative Enc34 endolysin, the coding sequence of the ORF39 protein with a cleavable N-terminal hexahistidine-tag was cloned and expressed in *Escherichia coli*. A plasmid construct encoding the full-length protein showed low transformation efficiency, reduced cell growth and no detectable production of the target protein upon induction, indicative of toxicity of the ORF39 gene product to the bacterial cells. Further experimentation revealed that a truncated variant of the ORF39 protein containing only the conserved PHA02564 domain can be readily produced in a soluble form and without adverse effects to the cells. This C-terminal deletion variant of the protein, ORF39ΔC, was therefore used for the subsequent studies.

To test for the enzymatic activity of the Enc34 endolysin, purified ORF39ΔC protein was subjected to turbidity reduction assays using outer membrane (OM)-permeabilized bacteria as a substrate. The Enc34 endolysin showed clear muralytic activity towards OM-permeabilized *Escherichia coli* W3100, *Pseudomonas aeruginosa* PAO1 and *Hafnia alvei* cells (Table 1), demonstrating that the N-terminal domain of the Enc34 endolysin, and hence the PHA02564 family proteins in other bacteriophage genomes, indeed encode an enzymatically active domain (EAD) of a functional endolysin. When using OM-permeabilized *P. aeruginosa* PAO1 cells, the enzymatic activity of the Enc34 endolysin was almost identical to commercial chicken egg white lysozyme but the activity was three to six times lower when using the other substrates. The ORF39ΔC protein showed no activity against unpermeabilized *E. coli* W3100, *P. aeruginosa* PAO1, or *H. alvei* cells (data not shown), suggesting that the EAD by itself cannot pass through the lipid bilayer, and it was also inactive against the Gram-positive microbe *Microbacterium paraoxydans*, which was unsurprising considering the notable differences in peptidoglycan structure between Gram-negative and Gram-positive bacteria^23^.

**Table 1:** Enzymatic activity of the phage Enc34 endolysin and comparison to chicken egg white lysozyme. *OM* outer membrane. Activity units and determination coefficients for the linear regression analysis are calculated according to^22^. Locally isolated and sequenced bacterial strain is indicated by an asterisk. Raw data can be found in the Supplementary Table S1.

Bacterial substrate	Lysozyme from chicken egg white (U/mg)	Determination coefficient R^2^	Enc34 endolysin, catalytic domain (U/mg)	Determination coefficient R^2^
OM-permeabilized *E. coli* W3100	13,170	0.9625	2250	0.9979
OM-permeabilized *P. aeruginosa* PAO1	16,890	0.9593	18,660	0.9846
OM-permeabilized H. alvei*	9330	0.9243	3180	0.9922

### Three-dimensional structure

The structure of the ORF39 EAD was initially determined using the single-wavelength anomalous diffraction method with selenomethionine-labeled ORF39ΔC crystals. The initial structure was solved to 1.8 Å which enabled building of an almost complete model of the EAD except for two poorly resolved loop regions. During subsequent crystal soaking and co-crystallization experiments with substrate analogs a different crystal form was discovered that diffracted to 1.6 Å. No electron density corresponding to the bound substrate could be identified in this structure, but due to the higher resolution and better resolved protein loops it was selected for reporting here.

**Figure 2:**
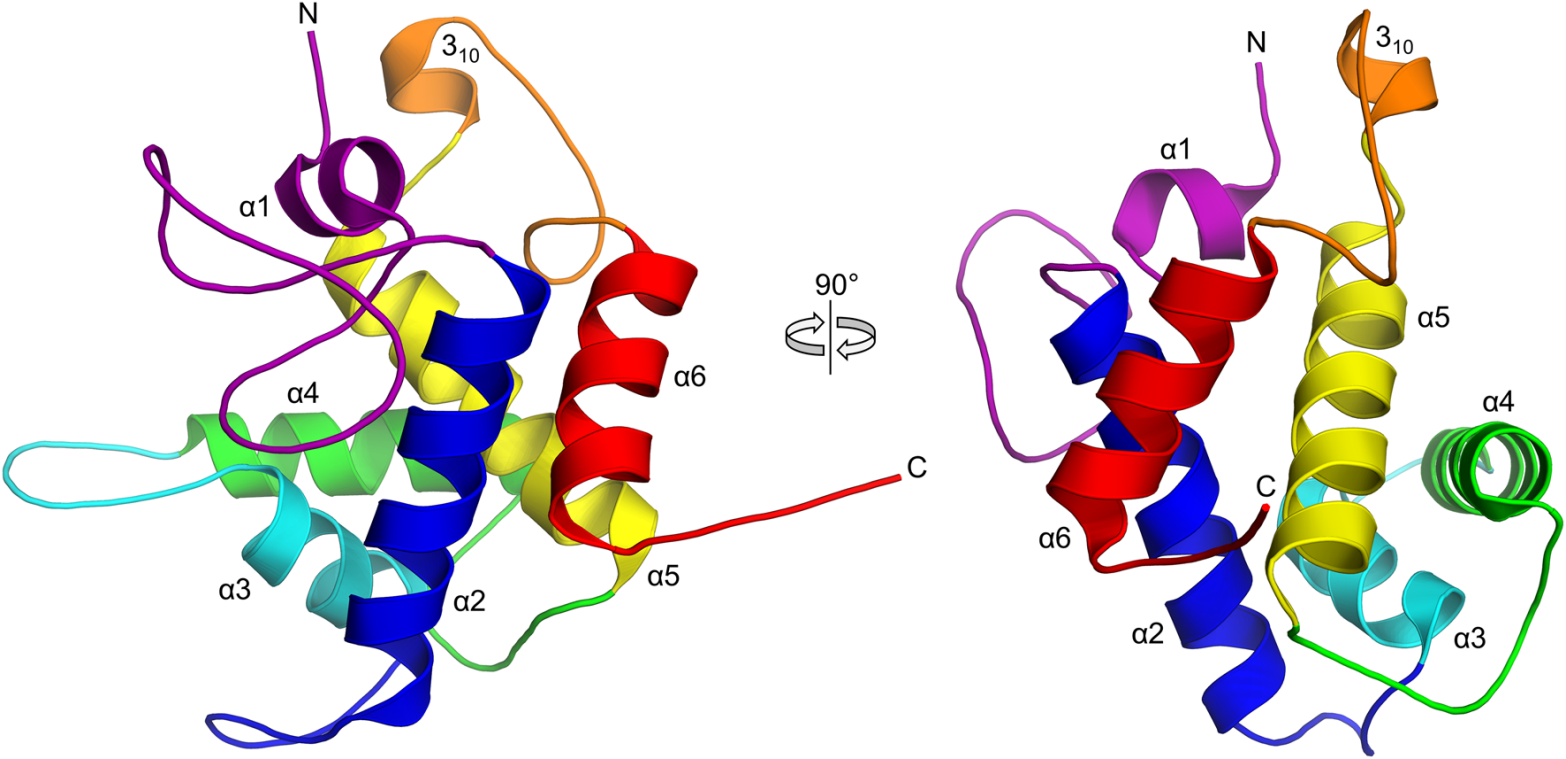
Three-dimensional structure of the Enc34 endolysin. The protein chain is rainbow-colored purple to red from the N- to the C-terminus. This and other structure figures were prepared using PyMOL^24^.

The crystallographic asymmetric unit contains two protein molecules which are represented as chains A and B in the final model. The two molecules have an interface area of 591 Å^2^ which due to its small size likely does not represent a biologically relevant assembly. Apart from three N-terminal residues that were poorly structured, the A monomer could otherwise be modeled without gaps whereas the model of the B monomer does not include residues 83–89 due to disorder. The overall structure of the ORF39 EAD (Fig. 2) is all-helical and consists of six α helices (α1 to α6) and a single one-turn 3_10_ helix in-between α5 and α6. The structure can be regarded as two overlapping helical bundles, the first of which is formed by α2, α3, α4 and α5 in a roughly antiparallel arrangement, and the second consisting of α2, α5 and α6 with α2 and α6 running parallel to each other. The overall shape of the EAD is roughly globular with a large groove between loops connecting α1–α2 and α3–α4 and centered on the C-terminal end of helix α3. The C-terminus of the ORF39ΔC protein extends away from the EAD and in the full-length protein likely functions as a linker to the separate transmembrane domain.

Structural homology analysis with DALI^25^ uncovered similarities with a wide range of α-helical proteins with neither the top hit (*Homo sapiens* mitochondrial dimethyladenosine transferase 1; PDB ID: 6AJK; Z-score: 4.8) nor many of the next structural matches having apparent evolutionary relatedness to endolysins. Only three proteins, namely, *N*-acetylglucosaminidase from *Thermotoga maritima* (PDB ID: 4QDN; Z score: 3.0), lytic transglycosylase gp144 from bacteriophage φKZ (PDB ID: 3BKH; Z score: 2.6), and the *E. coli* lytic transglycosylase MltC (PDB ID: 4C5F; Z score: 2.5) did indicate potential homology, however, the sequence similarity of Enc34 ORF39 to any of these proteins was too small for a reliable sequence alignment and thus for sequence-based identification of evolutionary conserved residues.

**Figure 3:**
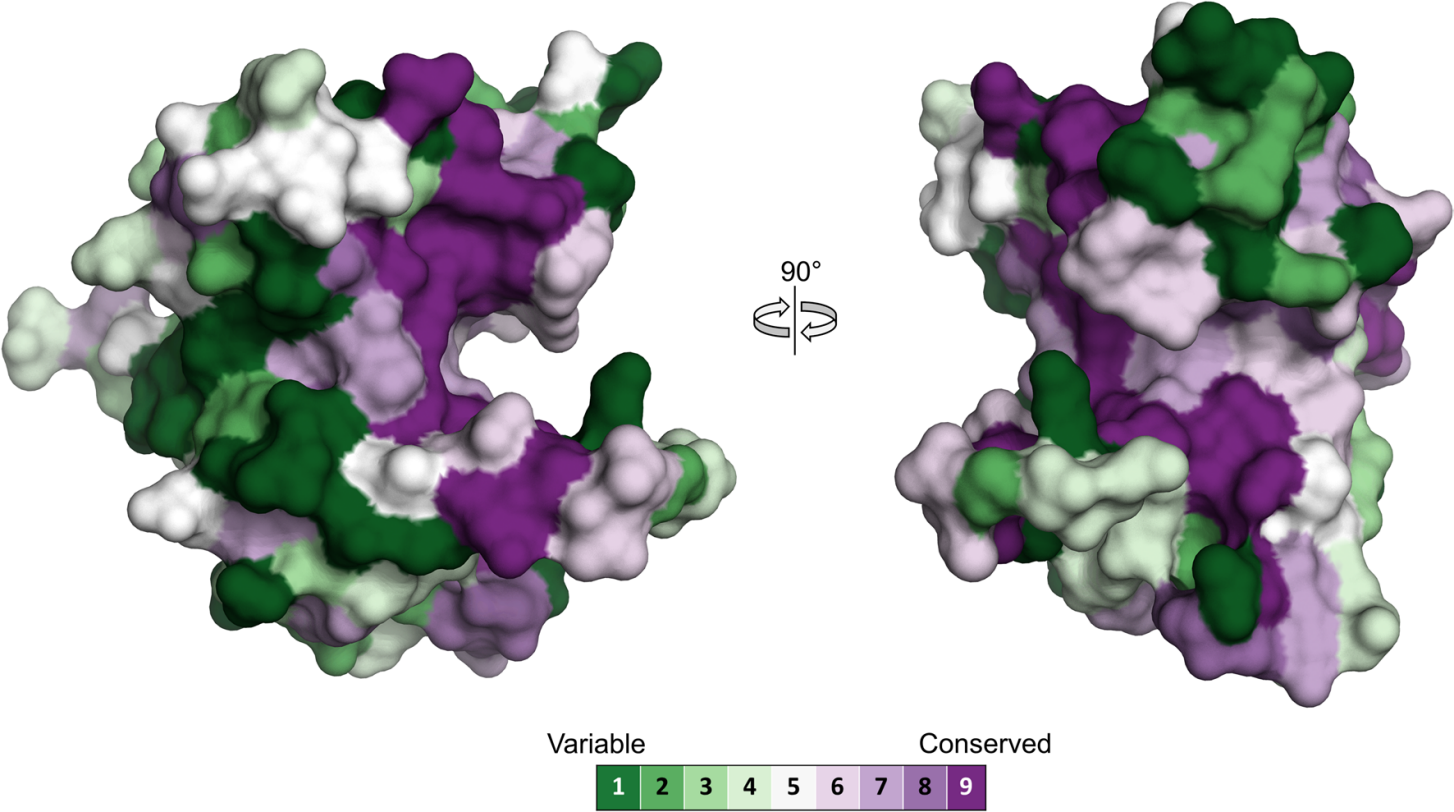
Amino acid conservation in the Enc34 endolysin. The analysis was performed on the ConSurf server^26^ with the default parameters for homolog search and multiple sequence alignment. The residues are colored according to the ConSurf conservation score (see legend).

Further conservation analysis with ConSurf^26^ revealed a trifurcated stretch of highly conserved residues centered around the major groove of the protein (Fig. 3) which probably constitutes the peptidoglycan binding surface of the enzyme. We were however unable to confirm carbohydrate binding to this area experimentally as crystal soaking and co-crystallization experiments with *N*-acetylglucosamine (NAG), *N*-acetylmuramic acid (NAM), chitotetraose or NAG–NAM disaccharide did not reveal bound sugar moieties in any of the trials, but the complex shape of the potential peptidoglycan-binding surface might indicate that the enzyme recognizes a branched motif within the peptidoglycan network which was not present in any of the tested substrates.

**Figure 4:**
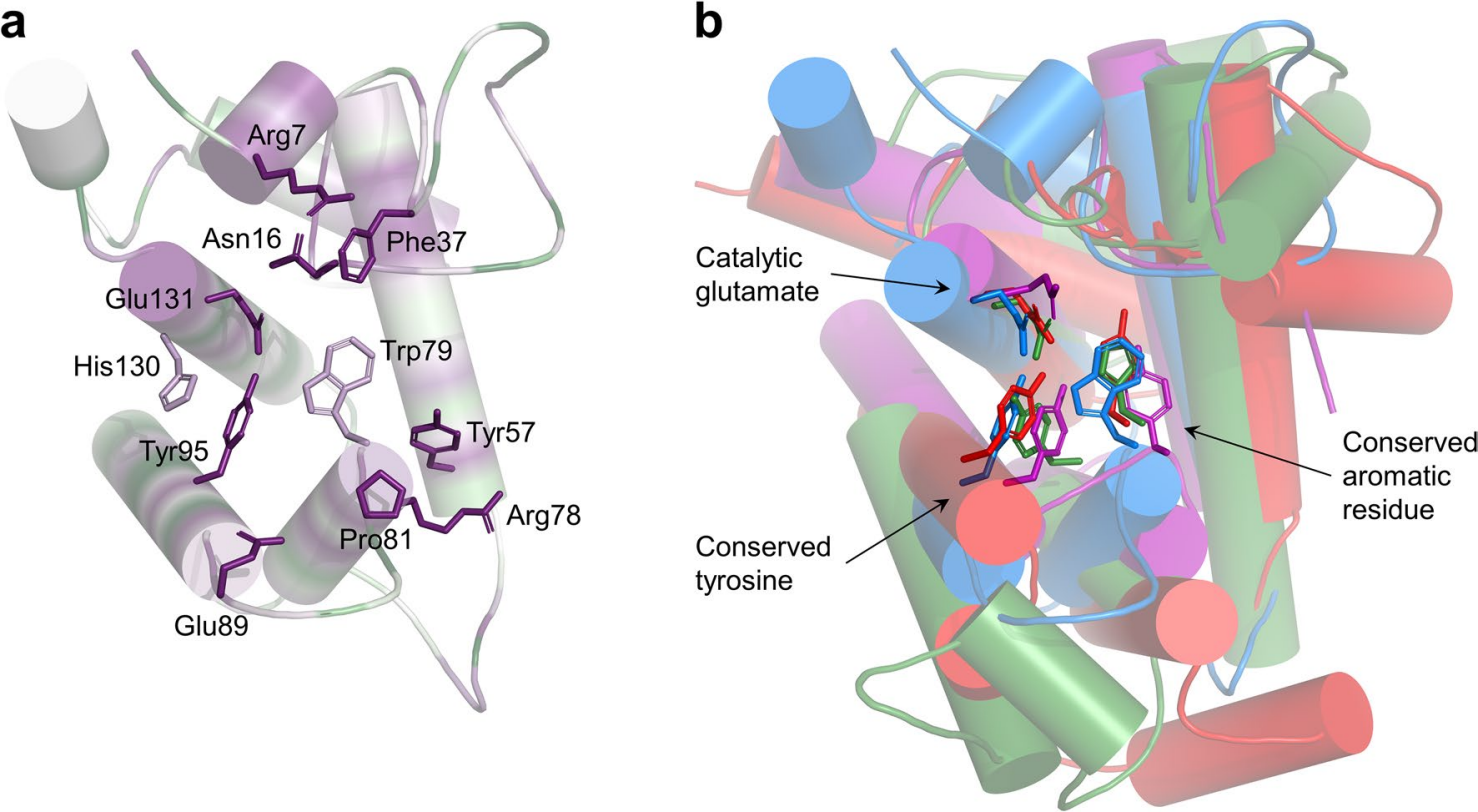
Substrate binding groove of the Enc34 endolysin and its structural homologs. (**a**) Conserved residues within the large groove of the Enc34 endolysin. The protein is colored as in Fig. 3. (**b**) 3D alignment of the Enc34 endolysin (blue), *Thermotoga maritima* TM0633 (magenta), phiKZ gp144 (green) and *Escherichia coli* MltC (red) proteins. The superimposition was performed by pairwise comparing the Enc34 EAD with the structural homologs using DALI^25^. Additional domains of the gp144 and MltC proteins have been removed for clarity.

Within the large groove of the ORF39 protein, a number of conserved charged, polar, and aromatic residues could be identified that are potentially involved in substrate binding and catalysis (Fig. 4a). The Glu131 in the ORF39 EAD corresponds to the catalytic glutamate residues in the superimposed TM0633, gp144 and MltC proteins (Fig. 4b) which suggests that the groove is indeed the active site of the Enc34 endolysin. Furthermore, residues corresponding to Trp79 and Tyr95 are also conserved; these have been shown to be important for catalysis in the structural homologs^27,28^, corroborating the evolutionary relatedness of these proteins.

## Discussion

The ORF39 protein of the Enc34 bacteriophage is in a number of ways unusual compared to the other currently characterized bacteriophage endolysins, starting with its uncommon two domain architecture. Typically, endolysins have either a simple globular structure, made up entirely by a single enzymatically active domain (EAD), or they are modular, consisting of one or several EADs and a cell wall-binding domain (CBD). The Enc34 endolysin is comprised of an N-terminal globular EAD linked to a C-terminal two-helix transmembrane domain (TMD) which is an uncommon arrangement that was first reported for the *Salmonella* phage Siskin^29^ and appears to be limited to a clade of bacteriophages of the corresponding Chivirus genus and some related viruses including Enc34. A superficially similar architecture is observed in signal-anchor-release (SAR) endolysins in which the EAD is preceded by an N-terminal transmembrane helix that functions as a signal sequence for translocating the EAD to the periplasmic space and anchoring the protein to the bacterial inner membrane. The TMD of the Enc34 endolysin, however, is located at the C-terminus of the protein and no known translocation mechanisms appear to exist that might be able to transport an N-terminal hydrophilic domain through the membrane using a C-terminal signal sequence. Still, recent studies of a homologous protein M4Lys from the *Salmonella* phage BSPM4^30^ have demonstrated that the Enc34-type enzymes appear to be capable of achieving bacterial lysis without the requirement for the holin protein which implies that the EAD does gain access to the peptidoglycan substrate through some unknown mechanism. The holin-independent lysis is apparently mediated by the TMD since both for the M4Lys and the Enc34 ORF39 only full-length proteins were toxic to the bacterial cells, and for M4Lys it was further established that expression of the C-terminal transmembrane helix alone results in a bacteriostatic effect. Some endolysins are known to have an intrinsic capacity to disrupt bacterial membranes by an amphipathic helix at the C-terminus^17,31^ or to cross the membrane using a cationic N-terminus^32^ but there are no good indications that any of these mechanisms apply to the Enc34-type endolysins which do not exhibit charged N-termini and, while the EAD-proximal TMD helix does appear to have amphipathic character, at least for the M4Lys protein expression of the EAD together with the proximal helix does not result in deleterious effects to cells.

Lysis timing in bacteriophage-infected cells is regulated by holin proteins which prevent endolysins from accessing the peptidoglycan substrate until the later stages of infection. The holins function either by forming large pores in the inner membrane that allow diffusion of endolysin molecules out of the cytoplasm or, in case of the SAR endolysins, by forming small “pinholes” which depolarize the membrane that in turn releases the anchored endolysins into the periplasm^33^. Due to the presence of the TMD it could be speculated that the Enc34-type endolysins are tethered and released from the inner membrane in a way functionally resembling the SAR endolysins and accordingly, the holin proteins in these viruses might function as pinholins. It can also be noted that the holin-independent lysis by the Enc34-type endolysins somewhat resembles observations with SAR endolysins^17,34^ but it is not entirely clear whether this phenomenon plays some role during the phage life cycle or it is merely a side effect of the strong promoter-driven recombinant expression system. Clearly, further studies of the holin-endolysin system of Enc34-like bacteriophages are required to address these questions experimentally.

Besides the perplexing TMDs, the EADs of the Enc34-type endolysins are equally distinctive. The Enc34 EAD does not have identifiable sequence similarity to proteins of known function, and only its three-dimensional structure is able to provide some clues about its relatedness to the other known classes of these enzymes. The closest structural homolog of the Enc34 endolysin, the protein TM0633 from the hyperthermophilic bacterium *Thermotoga maritima*^27^, is a member of the large glycoside hydrolase family 73 (GH73) which, according to the Carbohydrate-Active Enzymes Database (http://www.cazy.org), currently holds over 29,000 representative enzymes, while the two more distant matches, gp144 from bacteriophage phiKZ^35^ and MltC from *Escherichia coli*^36^, belong to the even bigger GH23 family with more than 100,000 constituent proteins. Proteins from these families span a considerable range of enzymatic activities that include 1,4-β-*N*-acetylmuramidases (EC 3.2.1.17), mannosyl-glycoprotein endo-β-*N*-acetylglucosaminidases (EC 3.2.1.96), peptidoglycan hydrolases with endo-β-*N*-acetylglucosaminidase specificity (EC 3.2.1.-), peptidoglycan lyases (EC 4.2.2.n1) and chitinases (EC 3.2.1.14). Structurally, however, these proteins are all variations of the α/β “lysozyme fold” which canonically consists of α- and β-structured parts (lobes) arranged to form a deep cleft within which the substrate binding and cleavage takes place. The EAD of the Enc34 endolysin bears resemblance to the α-lobe of the lysozyme fold but, in contrast to TM0633 and other GH73 enzymes, lacks any β-structured elements. It can be noted, however, that the large loop connecting α1 and α2 in the Enc34 ORF39 protein is located at essentially the same position as the β-lobe in GH73 enzymes and could likewise function as a lid over the substrate-binding groove.

**Figure 5:**
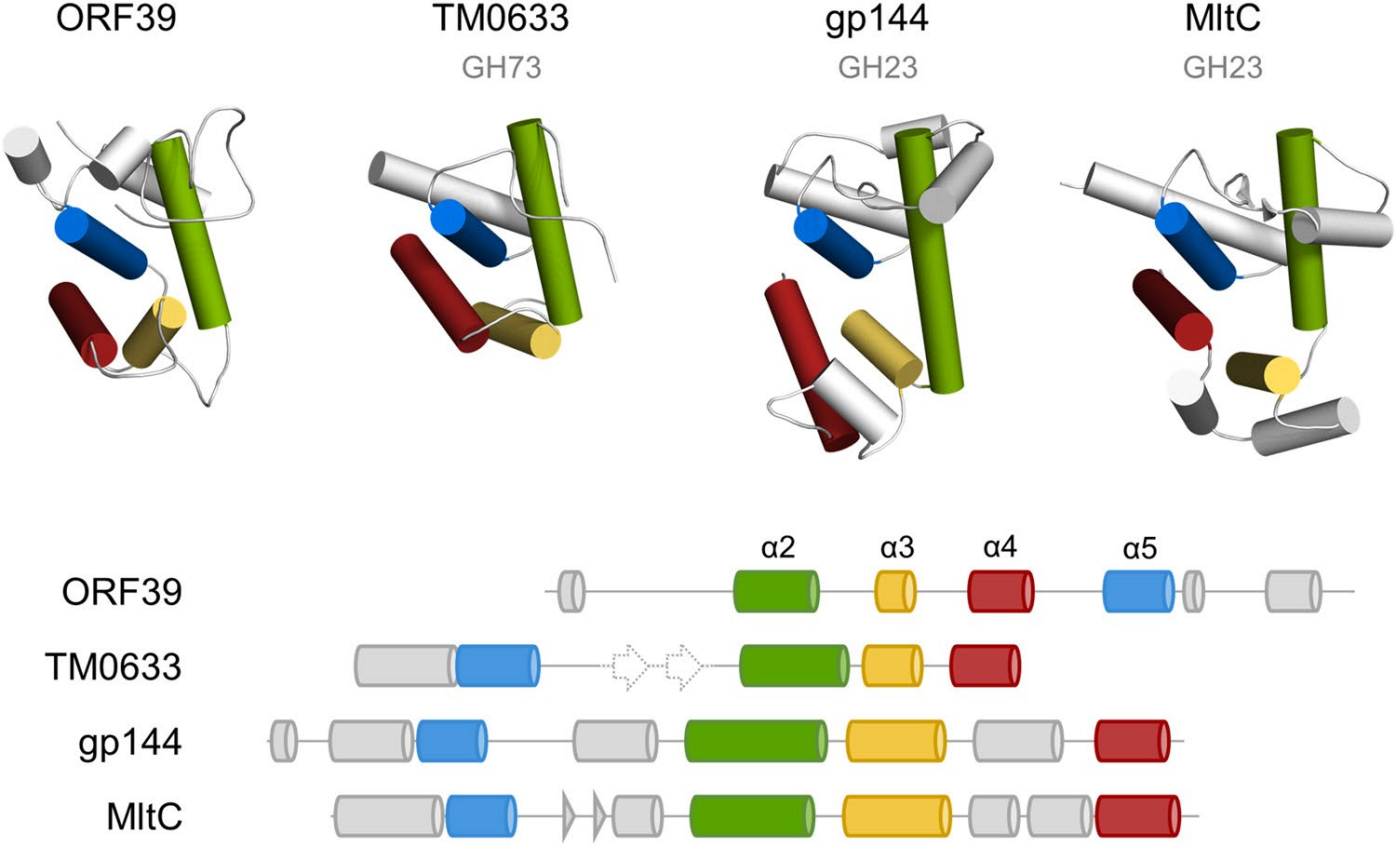
Similarities in the core fold of the Enc34 endolysin and GH73 and GH23 glycoside hydrolase enzymes. The Enc34 endolysin (ORF39), *Thermotoga maritima* TM0633, phiKZ gp144 and *Escherichia coli* MltC proteins are shown in the same orientation as in Fig. 4 with the four helices constituting the protein core shown in different colors. Secondary structure diagrams of the proteins are presented below using the same color scheme. A β-hairpin present in the TM0633 protein but not included in the model is shown in dashed representation.

All of the core helices α2, α3, α4 and α5 that form the major groove of the Enc34 endolysin have identifiable counterparts in the TM0633, gp144 and MltC proteins; however, despite similar three-dimensional arrangement, there is a marked difference in the sequential order of these helices between the Enc34 endolysin and the other proteins. Helices corresponding to α2, α3 and α4 in the Enc34 endolysin are found in that particular order also in the other structural homologs but while the remaining helix α5 in the Enc34 endolysin directly follows α4, in the TM0633, gp144 and MltC proteins the corresponding helix is located before their α2 counterpart (Fig. 5). Notably, the α5 holds the catalytic Glu131 of the Enc34 endolysin but despite the permuted core, the respective glutamate residue in the superimposed ORF39, TM0633, gp144 and MltC structures is located at essentially the same position as in the Enc34 protein (Fig. 4b). An equivalent tyrosine residue for Tyr95 and a matching aromatic residue for Trp79 can also be identified in the homologous GH73 and GH23 enzymes where these have been shown to be important for the enzymatic activity, potentially by correctly positioning the substrate in the active site^27,28,36^. Several other aromatic residues are found in vicinity to the active sites of these enzymes, a not uncommon characteristic for carbohydrate-binding proteins as the aromatic side chains can take part in both hydrogen bonding and non-polar CH-π interactions^37^. Overall, despite the unrecognizable sequence similarity and notable differences also in the three-dimensional structure, these conserved features indicate a shared evolutionary history between the Enc34 endolysin and GH73 and GH23 family proteins and highlight the potential minimum molecular requirements for peptidoglycan cleavage for this superclade of enzymes.

The bond specificity of the Enc34 endolysin was not experimentally investigated in this study but HPLC analysis of peptidoglycan digestion products by the related M4lys protein^30^ suggested both *N*-acetylglucosaminidase and endopeptidase activities for this enzyme. While two distinct enzymatic specificities for the same protein would appear unusual, the very low sequence similarity between the Enc34-type endolysins and any other characterized lytic enzymes left an open possibility that these proteins might represent a completely new class of enzymes with features that would explain such observations. The three-dimensional structure of the Enc34 endolysin has now revealed a conserved signature of three catalytically important residues and weak but recognizable similarity in the core protein fold that indicate that the Enc34-type enzymes represent a highly diverged lineage of glycoside hydrolase enzymes with common ancestry to GH73 and GH23 family proteins. The closest known structural homolog to the Enc34 endolysin, the *T. maritima* TM0633 protein, has been experimentally shown to be an *N*-acetylglucosaminidase but no endopeptidase activity has been reported for either the TM0633 protein or, to our knowledge, any other GH73 or GH23 enzyme. It therefore appears reasonable to conclude that the Enc34 endolysin, and by extension other proteins of the PHA02564 family, are β-*N*-acetylglucosaminidase (glycosidase) enzymes with a catalytic mechanism similar to that of GH73 family enzymes of the same bond specificity. We have not identified any specific clues within the three-dimensional structure of the Enc34 EAD which would suggest for the endopeptidase activity of this protein, but, although unlikely, it still cannot be excluded that Enc34-type enzymes have evolved a way to recognize and cleave two distinct substrates using essentially the same structural framework. Further dedicated studies would be required to explore such intriguing prospects in more detail and to gain deeper mechanistic understanding of these enzymes, for which the three-dimensional structure of the Enc34 endolysin should provide a valuable foundation.

## Methods

### Sequence analysis

Evolutionarily related proteins and conserved domains within the Enc34 lysis module were identified with BLASTP^38^ and HHpred^39^. Transmembrane helices were predicted with TMHMM^40^ and signal peptides were identified with SignalP^41^. To identify homologs of the Enc34 endolysin, the amino acid sequence of the ORF39 gene product (accession number: YP_007007038.1) was queried against sequences of viral origin (taxid: 10239) from the NCBI’s non-redundant protein sequence (nr) database with BLASTP using the default settings. Homologous sequences of comparable length (230–259 amino acids) were retrieved from the top 100 hits regardless their functional annotation except that metagenome-derived sequences were omitted, and a multiple sequence alignment of the resulting 37 sequences and the Enc34 endolysin was generated using Clustal Omega (v1.2.4)^42^ with the default settings. The resulting alignment with 301 columns (180 parsimony-informative, 39 singleton and 82 constant sites) was used to infer a maximum-likelihood phylogenetic tree with IQ-TREE (v2.0.6)^43^ using the LG + G4 substitution model (the best-scoring model according to the Bayesian information criterion as determined by ModelFinder^44^), allowing for polytomies and using 1000 ultrafast bootstrap replicates^45^ for assessing branch support. The resulting tree was midpoint-rooted and visualized in FigTree (v1.4.4)^46^.

### Protein production and purification

The coding sequence of the ORF39 protein was PCR-amplified from Enc34 genomic DNA (GenBank ID: JQ340774) using a phosphorylated forward primer 5′-CGCTAAGACGTCGTTGCCG-3′ and a reverse primer 5′-GTGCTTAAGTCATGCAGCCCCGGCCTTG-3′ for the full-length protein or 5′-GTGCTTAAGTCAAGTCTTTGGCTTAACCAATCC-3′ for the truncated protein containing only the conserved PHA02564 domain (residues 1–169). The amplified DNA was digested with *Bsp*TI (underlined) and cloned into a *Stu*I–*Bsp*TI digested pETDuet-1-derived vector^47^ that encodes an N-terminal hexahistidine-tag followed by tobacco etch virus (TEV) cleavage site for tag removal.

The ORF39ΔC protein was produced following a previously developed protocol^47^. Briefly, the ORF39ΔC expression plasmid was introduced into *Escherichia coli* BL21(DE3) cells and the bacteria were grown in 2xTY medium supplemented with 50 μg/mL ampicillin at 25 °C until OD_600_ of the culture reached 0.6–0.8, after which the growth temperature was reduced to 22 °C and IPTG was added to a final concentration of 0.01 mM to induce protein expression. After 16–18 h the cells were harvested by centrifugation, resuspended in TN buffer (20 mM Tris–HCl (pH 8.0), 300 mM NaCl), disrupted by sonication, and the lysate was clarified by centrifugation and applied onto a 1 mL HisTrap FF crude column (GE Healthcare). The column was washed with TN buffer containing 20 mM imidazole and the bound ORF39ΔC protein was eluted with TN buffer containing 300 mM imidazole. The eluted protein was digested with recombinant TEV protease overnight at 4 °C in presence of 1 mM DTT. The preparation was buffer-exchanged to TN using a 5 mL HiTrap Desalting column (GE Healthcare), passed through a HisTrap column and the flow-through containing the cleaved ORF39ΔC protein was collected.

To produce selenomethionine-substituted ORF39ΔC, *E*. *coli* B834(DE3) cells containing the ORF39ΔC expression plasmid were grown in 2xTY medium at 25 °C until OD_600_ of the culture reached 0.8–1.0. The cells were then centrifuged, resuspended and incubated in SelenoMet™ Medium Base supplemented with SelenoMet™ Nutrient Mix (Molecular Dimensions) at 25 °C for 2 h. Then, 1 × SelenoMethionine solution and 0.1 mM IPTG were added and the cultivation was continued overnight at 25 °C. The protein was extracted and purified following the same protocol as for the non-substituted ORF39ΔC, except that 5 mM DTT was added to the TN buffer and that all other buffer solutions contained 1 mM DTT to maintain reducing conditions.

### Turbidity reduction assay

The peptidoglycan substrate for the enzymatic assays was obtained by treating bacteria with chloroform-saturated 50 mM Tris–HCl (pH 7.7) as described previously^48^ and resuspending the sacculi in PBS at a concentration of OD_600_ of ~ 0.6–1.0. The *Escherichia coli* W3100, *Pseudomonas aeruginosa* PAO1, Enc34-sensitive *Hafnia alvei* and *Microbacterium paraoxydans* bacteria used in the study originated from laboratory collection. The enzymatic activity was assayed essentially as described previously^22^ by adding purified ORF39ΔC or chicken egg white lysozyme (Biochemica) in 30 μl of PBS to 270 μl of the sacculi stock in a 96-well plate and measuring changes in absorbance at 600 nm on a BioTek μQuant microplate reader for 3 h at 3 min intervals. Outer membrane-permeabilized cells were assayed using dilution series (0.1–5.0 μg) of the enzyme, each amount in triplicate, and the enzymatic activity was calculated using the ActivityCalculator tool (https://www.biw.kuleuven.be/logt/ActivityCalculator.htm)^22^. Untreated cells were tested only with the highest enzyme amount and compared to a negative control without the enzyme.

### Crystallization, data collection and structure determination

Purified ORF39ΔC or SeMet-ORF39ΔC protein was transferred to a buffer containing 20 mM Tris–HCl (pH 8.0), 100 mM NaCl and concentrated to ~ 10 mg/mL using Amicon 10 kDa MWCO filters (Millipore), and crystallized by mixing 1 μL of the concentrated protein solution with 1 μL of a solution containing 2.0 M ammonium phosphate monobasic and 0.1 M Tris–HCl (pH 8.5) using the sitting-drop vapor-diffusion technique. Crystals were flash-frozen in liquid nitrogen in a mother liquor containing 30% glycerol, and diffraction data from a native crystal diffracting to 1.8 Å resolution were collected at beamline 14.1 at BESSY II (Berlin, Germany) and from several selenomethionine-labeled crystals at MAX IV beamline BioMAX (Lund, Sweden). Diffraction images were processed with XDS^49^ through the XDSAPP^50^ interface, and multiple SeMet datasets were further scaled together with XSCALE^49^ to increase the anomalous signal. The structure was solved using the AutoSol wizard in Phenix^51^, and the resulting auto-built model was used to phase the higher-resolution native dataset, followed by manual model building in COOT^52^ and refinement in REFMAC^53^. In a subsequent co-crystallization screen with 100 mM *N*-acetylmuramic acid (Sigma-Aldrich), a crystal grew in different conditions (0.2 M sodium chloride, 0.1 M phosphate/citrate buffer (pH 4.2), 20% w/v PEG 8000). The crystal was flash-frozen directly from the drop and diffraction data were collected at MAX IV beamline BioMAX. The data were processed with XDS and the structure was solved by molecular replacement with PHASER^54^, followed by model completion in COOT and refinement with REFMAC. Quality of the final model was evaluated using MolProbity^55^. Data collection, scaling, refinement and model validation statistics are presented in Table 2.

**Table 2:** Crystallographic data collection, scaling, refinement and model validation statistics. Values in parentheses are given for the highest resolution bin. The Ramachandran plot, rotamer and clashscore statistics are according to MolProbity^55^.

**Data collection and scaling**	
Space group	P 21 21 2
Cell parameters	a = 65.30 Å b = 128.99 Å c = 36.28 Å
Wavelength (Å)	0.9763
Total number of observations	504,768
Number of unique reflections	41,857
Resolution (Å)	45.9–1.60
Highest resolution bin (Å)	1.70–1.60
Multiplicity	12.1 (10.7)
Completeness (%)	100.0 (100.0)
R_merge_	0.168 (1.525)
Mean I/σI	9.90 (2.03)
CC_1/2_	0.996 (0.768)
Wilson B-factor (Å^2^)	30.7
**Refinement**	
Resolution (Å)	45.9–1.60
Highest resolution bin (Å)	1.64–1.60
Number of reflections	
Work set	39,341
Free set	2071
R_work_	0.177 (0.300)
R_free_	0.209 (0.286)
Number of atoms	
Protein	2608
Water	264
Other	4
Average B-factor (Å^2^)	
Protein	25.2
Solvent	33.5
rms deviations from ideal geometry	
Bong lengths (Å)	0.009
Bond angles (°)	1.519
Ramachandran plot	
Residues in favored regions (%)	97.8
Residues in allowed regions (%)	100.0
Rotamers	
Favored (%)	96.4
Outliers (%)	0.00
Clashscore, all atoms	0.97

## Supplementary Information

Supplementary Table S1.

Supplementary Legend.

## Data Availability

Coordinates and structure factors for the reported structure are available from the Protein Data Bank under accession code 7Q47. Any other datasets supporting the conclusions of this article are available from the corresponding author on reasonable request.
