# Supplementary material for: Diversity of the lysozyme fold: structure of the catalytic domain from an unusual endolysin encoded by phage Enc34
Source: Sci Rep. 2022 Mar 23;12:5005. doi: 10.1038/s41598-022-08765-1 (PMC8943055; doi:10.1038/s41598-022-08765-1)
Supplement: Supplementary file 2 — Supplementary Legend. [file 41598_2022_8765_MOESM2_ESM.docx]

**Table S1.** Raw data for the enzymatic activity of the chicken egg white lysozyme and phage Enc34 endolysin.
